# Management of penile fracture with complete urethral transection in a resource‐limited setting: A case report

**DOI:** 10.1002/ccr3.9488

**Published:** 2024-10-18

**Authors:** Arens Jean Ricardo Médéus, Stevenson Saint Hubert, Mehul Sinha, Kohlz Erley Saint Jusca

**Affiliations:** ^1^ Department of General Surgery Hospital of the State University of Haiti Port‐au‐Prince Haiti; ^2^ Surgical Research and Global Education Lab (SURGE Lab) Port‐au‐Prince Haiti; ^3^ Department of Urology Hospital of the State University of Haiti Port‐au‐Prince Haiti; ^4^ Kasturba Medical College Mangalore India; ^5^ Faculty of Medicine and Pharmacy of the State University of Haiti Port‐au‐Prince Haiti

**Keywords:** penile fracture, surgery, trauma, urethral injury, urology

## Abstract

**Key Clinical Message:**

Penile fracture is a urological emergency that requires prompt surgical intervention for optimal outcomes. Early diagnosis based on clinical presentation and imaging if needed is crucial. Timely repair ensures the preservation of penile function and minimizes complications.

**Abstract:**

Penile fracture (PF) is a rare but significant urological emergency characterized by the rupture of the tunica albuginea, often resulting from blunt trauma during sexual intercourse or other activities. The typical presentation includes sudden and severe penile pain, an audible “cracking” sound followed by swelling and deformity. While PF generally does not involve the urethra, bleeding from the urethral meatus suggests an associated urethral lesion. Imaging such as ultrasonography or magnetic resonance imaging (MRI) can assess the extent of the injury. We report a case of a 28‐year‐old male who presented with sudden‐onset penile pain, swelling, and blood dripping from the urethral meatus after a traumatic sexual encounter. The injury occurred during vigorous intercourse when his erect penis struck his partner's buttocks. He heard a cracking sound followed by immediate detumescence, penile curvature, and urethral bleeding. At the hospital, the patient exhibited tachycardia and elevated blood pressure. Examination revealed deviation of the penis to the right, urethrorrhagia, and a tender, swollen penis. A clinical diagnosis of bilateral corpora cavernosa injury and partial rupture of the penile urethra was made. A urethral catheter was placed with difficulty, draining initially reddish urine followed by clear urine. Laboratory tests showed normal hemoglobin and coagulation profiles with mild bacteriuria and hematuria. Due to clear clinical signs, no radiological imaging was performed before surgery. Surgical exploration performed 48 h postinjury revealed a PF involving the right corpus cavernosum and a complete rupture of the spongy urethra. The corpus cavernosum was repaired using Vicryl 2–0 sutures followed by urethroplasty with Vicryl 5–0. Postoperative care included antibiotics and analgesics. The patient had an uneventful recovery with preserved penile sensitivity and function. PF is a rare urological emergency that necessitates prompt diagnosis and management to prevent long‐term complications. Although clinical presentation often suffices for diagnosis, imaging can be valuable in ambiguous cases. Surgical intervention is the preferred treatment, offering the best functional and cosmetic outcomes. This case underscores the importance of timely surgical repair to ensure favorable recovery and preservation of penile function.

## Graphical abstract

The image captures the critical phase of urethral realignment and repair during open surgery. Sutures are being placed on the urethra and corpora cavernosa, carefully restoring anatomical structures. This case illustrates the complexity of surgical intervention following penile fracture, highlighting the need for precise urethral repair to ensure functional recovery and avoid postoperative complications such as stricture or fistula formation.

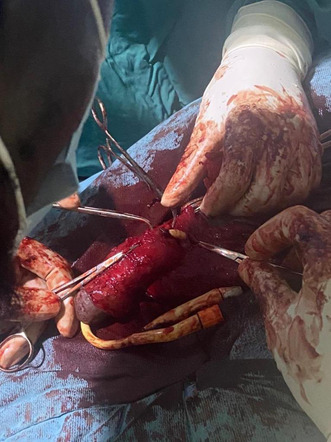

## 1 INTRODUCTION

Penile fracture (PF) is a rare but significant urological emergency with varying incidences across different regions. In the United States, the incidence is reported as 1.02 per 100,000 men per year, while in Iran, it is higher at 10.48 per 100,000 men.^1^ PF is characterized by the rupture of the tunica albuginea, the fibrous sheath surrounding the corpus cavernosum of the penis. It typically occurs as a result of blunt trauma during sexual intercourse, masturbation, or the practice of Taqaandan, which involves bending the erect penis for rapid detumescence.^1^, ^2^

Diagnosing PF primarily relies on clinical presentation, marked by sudden and severe penile pain, an audible “cracking” sound, and subsequent swelling and deformity. Generally, PF does not involve the urethra. However, if a patient with PF presents with bleeding from the urethral meatus, suspicion of an associated urethral lesion should be raised.^3^ To confirm the diagnosis and evaluate the extent of the injury, imaging studies such as ultrasonography or magnetic resonance imaging (MRI) may be utilized.^2^

Timely surgical intervention is the cornerstone of PF treatment, aiming to repair the tunica albuginea and restore penile integrity.^1^ Delayed or inadequate treatment can lead to complications such as permanent penile curvature, infection, and erectile dysfunction.^4^ Consequently, prompt diagnosis and appropriate management are crucial for achieving optimal outcomes in PF cases.

## 2 CASE HISTORY/EXAMINATION

A 28‐year‐old man presented to the urology department of the State University Hospital Haiti with sudden‐onset pain, a deformed and swollen penis, and blood dripping from the urethral meatus. He reported that the event occurred about 45 min before his arrival at the hospital during vigorous intercourse with his female partner in the doggy style position. His penis bumped against his partner's buttocks, and he heard a cracking sound followed by extreme pain, immediate detumescence, curvature of the penis, and blood at the urethral meatus. The patient had no known comorbidities or past surgical history. He arrived at the department a little panicked and worried with tachycardia of 140 bpm, blood pressure of 150/100 mm Hg, and a respiratory rate of 31 cycles per minute. Other vital signs were normal.

On local examination, the penis was deviated to the right at the proximal one‐third and distal two‐thirds junction. Urethrorrhagia and an increase in volume at the root of the penis were noted, and the penis was tender on manipulation. After evaluation, a clinical diagnosis of bilateral corpora cavernosa injuries and partial rupture of the penile urethra was made. A urethral catheter was placed with difficulty and initially drained reddish urine followed by clear urine. While waiting for laboratory tests, he received analgesics, and cold compresses were applied to the penis. A blood count showed a hemoglobin level of 15 g/dL and coagulation profile, and chlamydia serology were within normal limits. A urine examination showed 7–9 bacteria per field, 4–6 leukocytes per field, and 9–11 red blood cells per field. Radiological images were not obtained prior to surgical intervention.

## 3 METHODS (DIFFERENTIAL DIAGNOSIS, INVESTIGATIONS, AND TREATMENT)

### 3.1 Differential Diagnosis

- Penile fracture without urethral injury
- Penile fracture with complete urethral disruption
- Penile soft tissue injury

### 3.2 Investigations

- Clinical evaluation confirmed the diagnosis of PF with urethral injury. Given the clear clinical signs, radiological imaging was deemed unnecessary prior to surgical intervention.

### 3.3 Treatment

**FIGURE 1:**
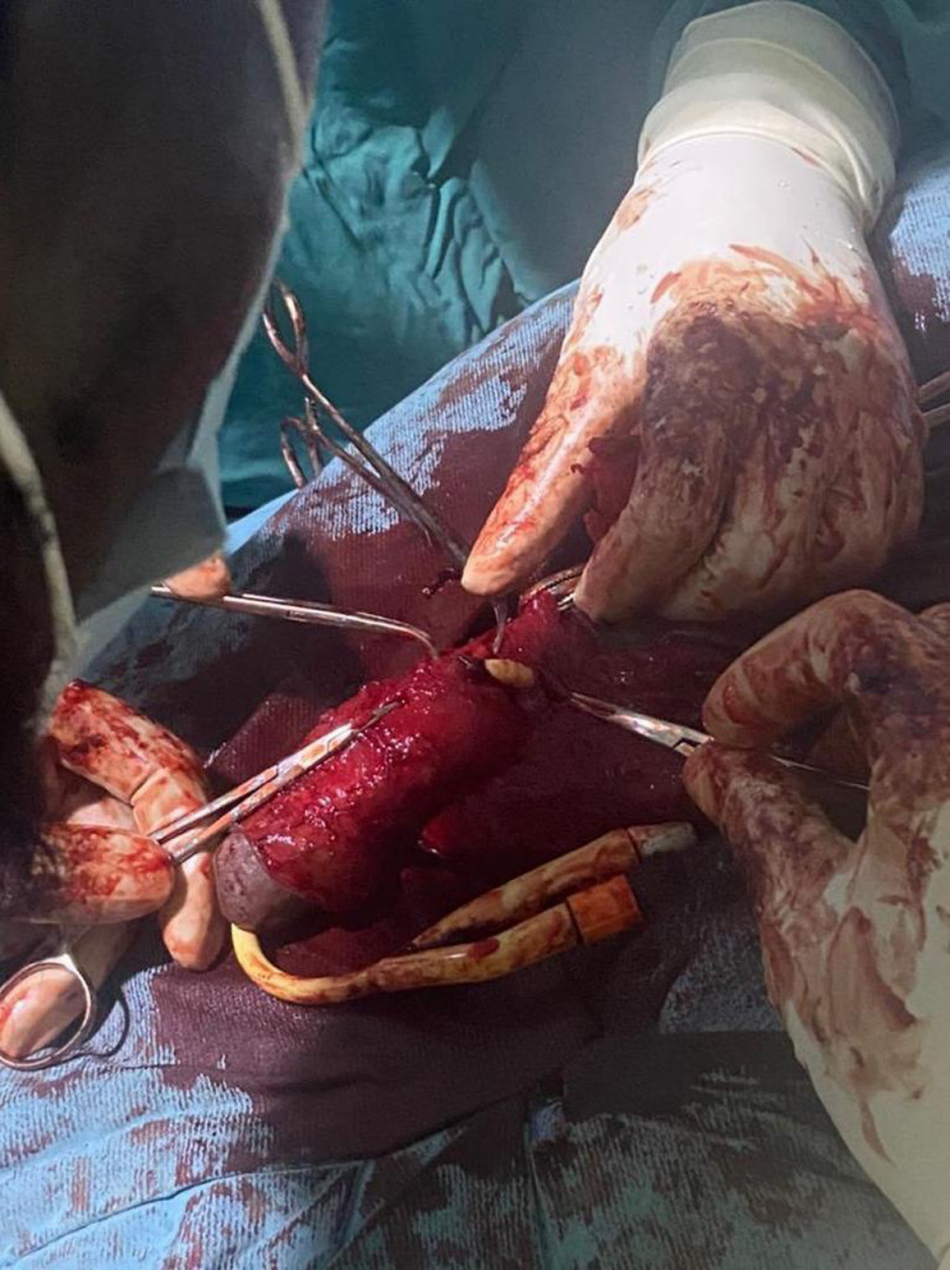
Complete section of the urethra with visualization of the urethral catheter connecting the two ends of the urethra.

Forty‐eight hours later, a surgical exploration of the penis was scheduled. First, the frenulum was ligated and sectioned. An inverted V incision was made from the root of the frenulum, and a circular incision one finger's breadth from the balano‐preputial fold was followed by complete dissection of the prepuce down to the root of the penis. Given the increased volume at the root and the distal location of the rupture, a longitudinal incision on the ventral surface of the prepuce was made to better visualize the lesion site. Intraoperatively, it was concluded that it was a PF with an injury of the right corpus cavernosum associated with a complete disruption of the spongy urethra (Grade IV AAST urethra injury scale) (Figure 1). The corpus cavernosum was repaired with Vicryl 2–0 followed by urethroplasty with Vicryl 5–0 then repair of the albuginea with Vicryl 2–0. The excess foreskin was removed and Vicryl 2–0 was used for hemostasis before suturing the skin with chromic 3–0.

Postoperative care included broad‐spectrum antibiotic therapy with a third‐generation cephalosporin; analgesics and a proton pump inhibitor (PPI) were also added.

## 4 CONCLUSION AND RESULTS (OUTCOME AND FOLLOW‐UP)

**FIGURE 2:**
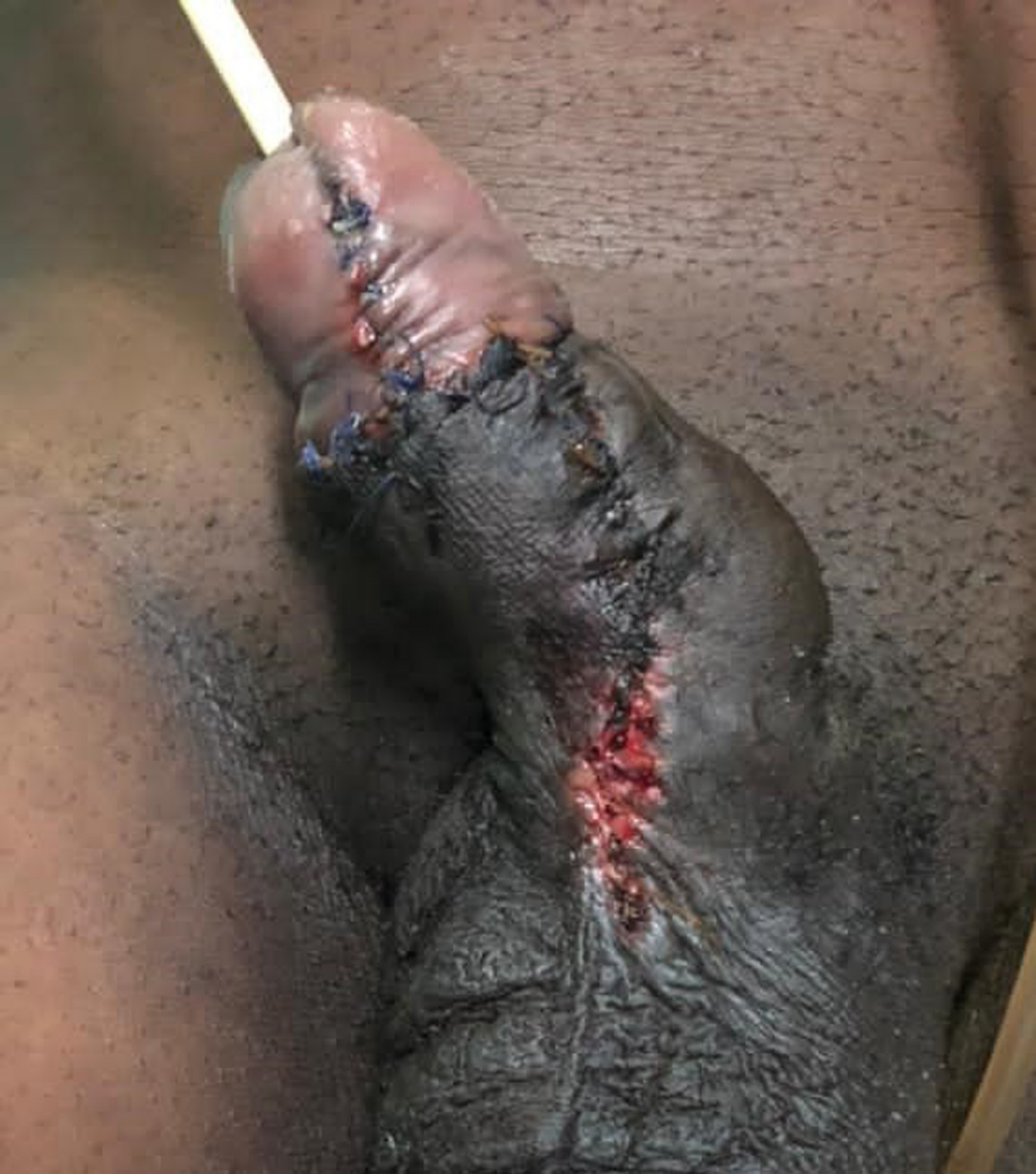
Operative wound on postoperative day 7.

**FIGURE 3:**
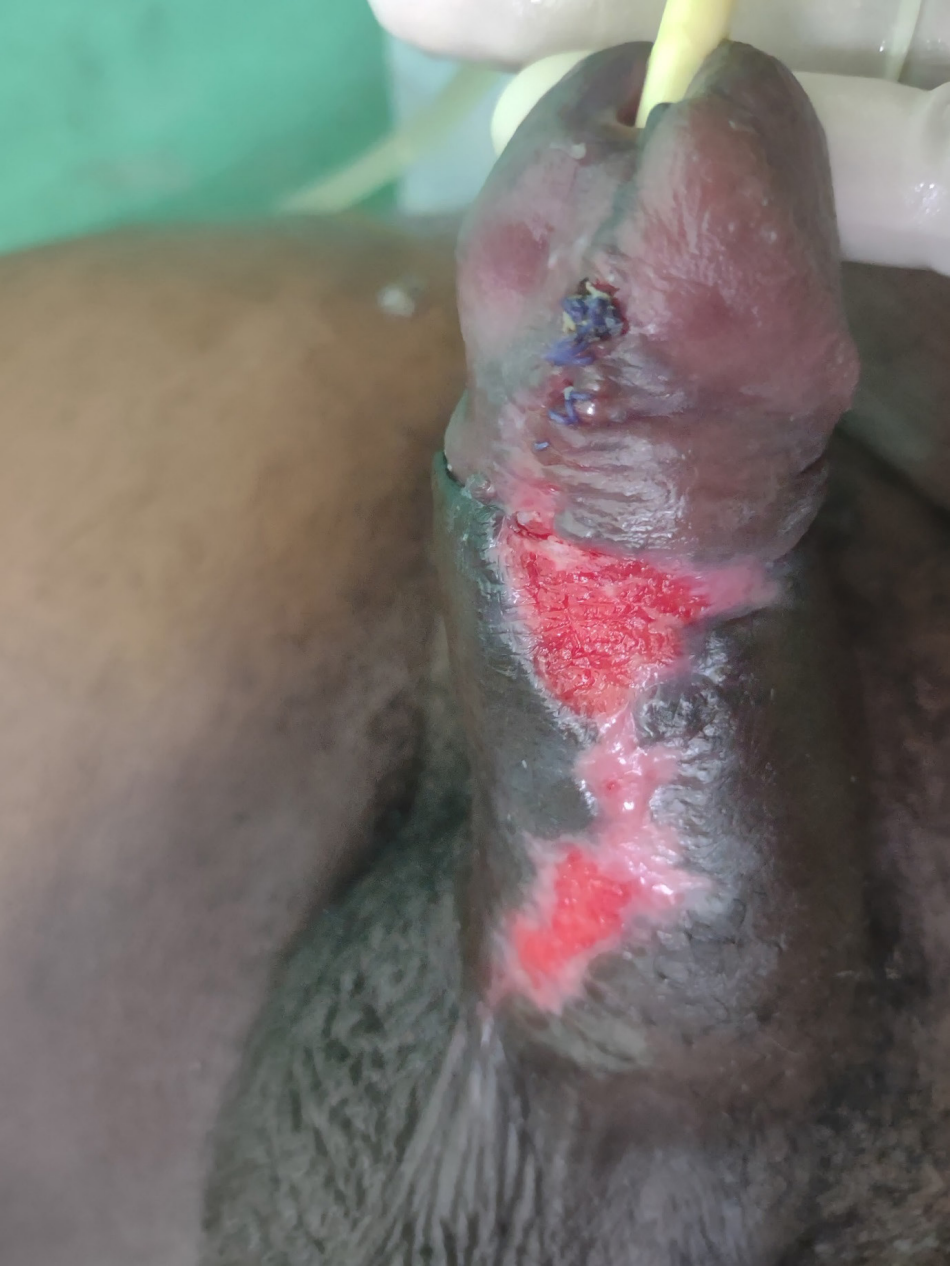
Operative wound on postoperative day 22.

The patient's postoperative course was uneventful. The surgical wound healed very well (Figure 2, Figure 3). He preserved penile sensitivity and experienced nocturnal erections soon after surgery. He was discharged after 2 days with instructions to keep the urethral catheter for 6 weeks and perform regular wound care. No complications were observed during follow‐up visits.

## 5 DISCUSSION

Penile fracture is a traumatic rupture of the tunica albuginea, considered a urological emergency.^5^, ^6^ The penis consists of the glans, a pair of corpora cavernosa surrounded by skeletal muscle and the tunica albuginea, and the corpus spongiosum with the bulb of the penis.^7^ The corpora cavernosa and corpus spongiosum are erectile tissues covered with fascia: the innermost being the tunica albuginea, the middle Buck's fascia, and the superficial Dartos fascia. When the penis becomes erect, the two corpora cavernosa engorge with blood, and the tunica albuginea becomes rigid, preventing venous return to maintain tumescence.^8^

The tunica albuginea has an average thickness of 2.4 mm,^9^ composed of collagen and elastin, with a remarkable tensile strength that can withstand an increase in intracavernosal pressure of over 1500 mmHg.^10^ When the collagen and elastin fibers are subjected to a force greater than their maximum tensile strength, they rupture.^9^ The thick tunica albuginea thins significantly from 2.4 mm to 0.25–0.5 mm as the penis transitions from a flaccid to an erect state. During this process, it becomes stiffer, loses elasticity, and is consequently more susceptible to fracture.^5^ Any firm trauma or sudden sideways force on the penis in this condition can cause the tunica albuginea to rupture.^6^, ^8^

The main circumstances of PF occurrence reported in a meta‐analysis include sexual intercourse (46%), forced flexion (21%), masturbation (18%), rolling over (8.2%), and other activities.^11^ Most often, the involvement is unilateral and, on the right, as confirmed in our case after surgery.^12^

Patients often complain of hearing a cracking or snapping sound, accompanied by a sudden loss of erection and pain.^8^ Physical examination reveals a swollen, ecchymotic penis deviated to the healthy side, curved in a tight S‐shape, known as the eggplant deformity or aubergine sign.^6^, ^8^, ^10^ The lesion site can be palpated where the hematoma is formed, known as the rolling sign.^8^, ^10^

The urethra may be affected, presenting with hematuria, blood in the urinary tract, or an inability to urinate.^6^ Urethral involvement is rare and tends to accompany bilateral fractures.^12^ In our case, the complete urethral rupture was associated with the involvement of the right corpus cavernosum. Amer et al. found urethrorrhagia in 5.6% of cases, while urethral involvement was confirmed in 6.1%. This indicates that the passage of blood into the urinary meatus is an indicator of urethral rupture, but its absence does not rule it out.^11^

In most cases, a rigorous clinical examination alone is sufficient to diagnose a PF.^1^ Imaging is only used in equivocal cases to locate the site of rupture or exclude a probable urethral injury.^13^ In our case, the patient presented the typical signs of a PF.

The most commonly used imaging tests are sonography and MRI. Sonography is inexpensive, rapidly and easily available, and noninvasive, making it the preferred choice of clinicians.^1^ It aids in diagnosis by enabling the localization of tears and hematomas, with a sensitivity of 88%, compared to MRI's sensitivity of 100% and a localization accuracy of 97.^1^, ^14^ Sonography is useful in confirming the diagnosis but does not necessarily influence the course of surgery. MRI can detect not only damage to the corpora cavernosa, but also sections of the corpus spongiosum.^15^ MRI, though ideal for diagnosis, localization, and follow‐up, is limited by its high cost and availability.^15^ The guidelines of the European Association of Urology mention other less commonly used options such as cavernography, retrograde urethrogram, or cystoscopy if urethral involvement is suspected.^16^ Nevertheless, the use of imaging has not proved necessary in our case, given the typical presentation of the signs. Moreover, most of these imaging studies like MRI are not available in our institution.

Management of PFs can be conservative or surgical.^17^ In the past, management was conservative^18^ until 1936 when the surgical method was described as the surgical method for the first time by Felter and Gartmen.^19^ The conservative method can be summed up as rest while applying hot or cold compresses, wearing tight underwear, nonsteroidal anti‐inflammatory drugs (NSAIDs) and penoscrotal elevation, prophylactic antibiotic therapy and sometimes the use of anti‐erection agents.^20^ This method is associated with a higher complication rate, is only considered in patients with partial tears or minor clinical signs, though it often leads to unsatisfactory outcomes.^1^ Surgical intervention remains the gold standard for managing PFs.^21^ In cases of urethral injury, the primary objectives are to preserve sexual potency and restore normal urinary function.^22^ These injuries may be partial or complete, and repair typically involves a tension‐free end‐to‐end anastomosis around a 16 or 18 French transurethral catheter using 4–0 or 5–0 absorbable sutures.^22^, ^23^ The transurethral catheter should remain in place for at least 14 days for partial lesions and up to 21 days for complete lesions.^24^ For complete ruptures or unstable patients, the addition of a suprapubic catheter is recommended, which should be kept closed for at least 3 days after removal of the transurethral catheter to ensure normal micturition before removal.^22^ Some clinicians suggest interposing a small amount of viable tissue between the urethra and the corpora cavernosa to prevent fistulas, which, if they occur, should be treated conservatively with a urethral catheter for approximately 30 days.^24^, ^25^

The choice of incision technique depends on the fracture's location and includes various approaches,^26^ such as the subcoronal circular incision, which provides access to the corpora cavernosa and spongiosa along the entire length of the penis and allows for the separation of the vascular‐nervous bundle if necessary. Other techniques include the medioventral penoscrotal incision, the lateral incision, and the inguinoscrotal incision.^1^

Timeliness of repair plays a crucial role in patient prognosis. Studies indicate that prompt surgical management reduces hospitalization time, presents fewer complications, and leads to better outcomes,^23^ particularly regarding erectile function.^27^ In our case, the patient experienced morning erections of short duration less than 7 days postoperatively.

Common complications include palpable penile nodules (13.1%), penile curvature (2.8%), erectile dysfunction (1.9%), painful erections (1.4%), and wound infections (0.2%).^1^

Finally, performing circumcision if not already done is advisable postsurgery to avoid postoperative phimosis. Patients should be recommended to abstain from sexual activity for at least 6 weeks to prevent potential dehiscence of the corpora cavernosa.^1^, ^25^

Our case involved a patient with clinical and paraclinical signs suggestive of urethral rupture, confirmed during surgery. Conservative management was not an option, given the extent of the injury. Studies have shown that surgical intervention offers better long‐term outcomes, with a low risk of erectile dysfunction or penile curvature. In our case, surgery was crucial to repairing the tunica albuginea and urethra, preventing potential complications such as fistula, stricture, erectile dysfunction, or penile curvature. However, for our patient the operation was delayed by 48 h due to logistical issues, including limited availability of operating room resources and essential surgical supplies at our institution. However, during this interim period, the patient was managed conservatively with initial care including pain relief, cold compresses and catheterization, which ensured that his condition remained stable until surgery could take place. Postsurgical management includes antibiotic therapy, NSAID's, catheter placement for 6 weeks, and follow‐up for complications. The patient recovered well, with preserved penile sensitivity and function, and no reported complications.

## AUTHOR CONTRIBUTIONS

**Kohlz Erley Saint Jusca:** Formal analysis; investigation; project administration; validation; visualization; writing – original draft; writing – review and editing. **Arens Jean Ricardo Médéus:** Conceptualization; data curation; formal analysis; project administration; supervision; validation; visualization; writing – original draft; writing – review and editing. **Mehul Sinha:** Data curation; formal analysis; validation; writing – original draft; writing – review and editing. **Stevenson Saint Hubert:** Data curation; formal analysis; investigation; validation; visualization; writing – original draft; writing – review and editing.

## FUNDING INFORMATION

We received no funding for this study.

## CONFLICT OF INTEREST STATEMENT

The authors declare that they have no competing interests.

## ETHICS STATEMENT

This study was conducted in accordance with the ethical standards laid down in the 1964 Declaration of Helsinki and its later amendments. According to the policies of Haitian State University Hospital, IRB approval was not required for this case report.

## CONSENT

Written informed consent was obtained from the patient to publish this report in accordance with the journal's patient consent policy.

## Data Availability

All data generated or analyzed during this study are included in this published article (and its supplementary information files).
